# circMRPL35 promotes gastric cancer progression through the miR-6809-3p/ZNF90 axis and affects the EMT process and TGF-β1/SMAD2/3 signaling

**DOI:** 10.1016/j.ncrna.2025.10.002

**Published:** 2025-10-10

**Authors:** Xiuping Wang, Zhendong Yao, Yu Liu, Boneng Mao, Chen Shao, Shihe Shao

**Affiliations:** a Department of Clinical Laboratory, Affiliated Kunshan Hospital of Jiangsu University, Kunshan, Jiangsu, 215300, China; b Jiangsu Key Laboratory of Medical Science and Laboratory Medicine, School of Medicine, Jiangsu University, Zhenjiang, Jiangsu, 212013, China; c Department of Gastroenterology, The Affiliated Yixing Hospital of Jiangsu University, Yixing, Jiangsu, 214299, China; d School of Medicine, Jiangsu University, Zhenjiang, Jiangsu, 212013, China; e Department of Cardiology, Affiliated Hospital of Jiangsu University, Zhenjiang, Jiangsu, 212013, China

**Keywords:** Gastric cancer, circMRPL35, miR-6809-3p, ZNF90, TGF-β1/SMAD2/3

## Abstract

**Background:**

Circular RNAs (circRNAs) have been implicated in developing gastric cancer (GC). However, the role of circMRPL35 in GC remains unknown.

**Methods:**

This study identified differentially expressed circMRPL35 using gene expression profiles GSE78092, GSE131414, and GSE100170. RNA R enzyme and RNA FISH assays were used to explore the origin, cyclization site, and cellular localization of circMRPL35. The functions of circMRPL35, miR-6809-3p, and ZNF90 in GC cells were evaluated through loss- and gain-of-function experiments. The epithelial-mesenchymal transition (EMT) process and the TGF-β1/SMAD signaling pathway were analyzed using Western blot and immunofluorescence assays. Subcutaneous tumor models in nude mice were utilized to evaluate the impact of circMRPL35 on GC cells. The interactions among circMRPL35, miR-6809-3p, and ZNF90 were confirmed through luciferase reporter and rescue assays.

**Results:**

The study demonstrated that circMRPL35, present in the cytoplasm and nucleus of MGC-803 and HGC-27 cells, originates from the cyclization of exons 4 and 5 on chromosome 2. In GC tissues and cells, circMRPL35 and ZNF90 were upregulated, while miR-6809-3p was downregulated. circMRPL35 and ZNF90 enhanced cell mobility and invasion and suppressed apoptosis by modulating the EMT process and TGF-β1/SMAD2/3 signaling pathway; conversely, miR-6809-3p exhibited the opposite effects. Mechanistically, circMRPL35 sponges miR-6809-3p to regulate ZNF90, thereby enhancing the phenotype of GC cells.

**Conclusions:**

These results indicate that circMRPL35 acts as an oncogenic driver via the miR-6809-3p/ZNF90 axis, affecting the EMT process and the TGF-β1/SMAD2/3 signaling pathway to promote GC progression.

## 1 Introduction

Gastric cancer (GC) ranks as the fourth leading cause of cancer-related deaths, presenting as a prevalent digestive tumor with a high incidence rate [1]. Most patients with low early diagnosis rates typically have poor prognoses at advanced stages and miss the best surgical window once diagnosed [2]. Thus, developing detection methods and identifying genetically specific biomarkers are essential for early diagnosis of GC. The complex process of GC involves environmental, host, and polygenic factors, which may offer insights for early detection or treatment [3].

Circular RNAs (circRNAs) are a unique type of noncoding RNAs resulting from back-splicing pre-mRNAs, forming a closed loop structure with a 3′ head and a 5′ tail [[4], [5], [6]]. CircRNAs exhibit more excellent resistance to RNase R and longer half-lives than linear RNA. They are widely distributed in zooblasts and show promise as diagnostic biomarkers and therapeutic targets [7]. CircRNAs can interact with RNA binding proteins (RBPs) [8], regulate parental gene transcription, facilitate protein translation [9], and act as competitive endogenous RNAs (ceRNAs) by sequestering microRNAs (miRNAs) [10,11]. The ceRNA mechanism is extensively studied for its role in gene expression regulation, particularly in tumor progression affecting cellular processes like the cell cycle, apoptosis, proliferation, migration, and invasion in various malignancies [[12], [13], [14]], notably in GC [15]. Investigating the mechanistic role of circRNAs in GC can enhance our understanding of the disease and provide a solid foundation for developing diagnostic markers.

This study discovered a GC-related circRNA, circMRPL35, originating from exons of MRPL*35* and identified with a circBase ID of hsa_circ_00055521. Both GC tissues and cell lines exhibited upregulation of circMRPL35. Notably, circMRPL35 acts as a sponge for miR-6809-3p, alleviating its inhibition of ZNF90 to regulate the TGF-β1/SMAD signaling pathway involved in the EMT process. This mechanism leads to enhanced migration and proliferation of GC cells and suppression of apoptosis. The results indicate that circMRPL35 functions as an oncogene in GC progression through the miR-6809-3p/ZNF90 axis, suggesting its potential utility as a diagnostic marker and therapeutic target for GC patients.

## 2 Methods

### 2.1 Cell culture

GC cell lines, including HGC-27, MGC-803, SGC-7901, BGC-823, human normal gastric mucosal cells (GSE-1) and HEK-293T cells, were purchased from the Chinese Academy of Sciences Affiliated Cell Resource Center of Shanghai Institute of Life Sciences (Shanghai, China) in 2020 and cultured in Dulbecco's modified Eagle's medium (DMEM; Gibco, Grand Island, NY, USA) supplemented with 10 % fetal bovine serum (FBS; Life Technologies, USA), 1 % streptomycin and penicillin (Gibco) at 37 °C, 5 % CO_2_, and saturated humidity.

### 2.2 Western blot

Total proteins were extracted using radioimmunoprecipitation assay buffer (RIPA) supplemented with 1 % phenylmethanesulfonyl fluoride (PMSF; Beyotime), followed by separation using SDS-PAGE and transfer onto polyvinylidene fluoride (PVDF) membranes (Millipore, MA, USA). Subsequently, the PVDF membranes were blocked with 5 % skim milk for 2 h, incubated overnight at 4 °C with the respective primary antibodies, and then incubated for 1 h at room temperature with secondary antibodies. The Image Quant LAS 4000 mini (Pittsburgh, PA, USA) enhanced chemiluminescence (ECL) system was employed for membrane analysis.

### 2.3 Transfection of oligonucleotides and plasmids

Oligonucleotides (GenePharma) and plasmids (Sangon Biotech, Shanghai, China) were designed and produced to modulate the expression of circMRPL35, miR-6809-3p, and ZNF90. The oligonucleotides and plasmids were transfected into cells using Lipofectamine 8000 (Beyotime, Shanghai, China) following the manufacturer's instructions. The specific sequences can be found in Table S1.

### 2.4 RNA isolation, RNase R treatment, and quantitative real-time PCR (qRT‒PCR)

Total RNA was extracted using TRIzol (Vazyme, Nanjing, China) following the manufacturer's protocol. To treat with RNase R, 10 μg of total RNA was incubated with 20 U of RNase R (BioVision, Palo Alto, USA) at 37 °C for 15 min. To treat with RNase R, 10 μg of total RNA was incubated with 20 U of RNase R (BioVision, Palo Alto, USA) at 37 °C for 15 min cDNA was synthesized from RNA through reverse transcription with HiScript® III 1st St (Novozymes, Nanjing, China). Relative expression levels were determined using the 2^−ΔΔ*C*t^ method, with GAPDH or U6 as the internal controls. Specific primer details can be found in Table S2.

### 2.5 Cell counting Kit-8 (CCK-8) proliferation and colony formation assay

The CCK-8 test was performed to evaluate the transfected cells' viability. Specifically, 96-well plates were utilized, with 1000 cells per well and three duplicates for each treatment. The optical density was assessed using a microplate reader (Biotek, USA), with absorbance readings taken at 450 nm. Six-well plates were used for the colony formation assay, each with three replicates and approximately 1000 transfected cells per well. Following a two-week incubation period, the cell clones were fixed using 4 % paraformaldehyde and then stained with crystal violet (Chemical Reagent, Nanjing, China).

### 2.6 Transwell migration assay

The transwell upper chambers (Bio-Rad, CA, USA) were seeded with 1 × 10^5^ transfected cells in 300 μL serum-free media, and the bottom chambers were filled with 600 μL fresh medium containing 10 % FBS. Transwell top chambers were cultured for 24 h before being cleaned with phosphate-buffered saline (PBS), fixed with 4 % paraformaldehyde, and stained with crystal violet. Finally, the cells were counted and photographed under a microscope.

### 2.7 Cell wound healing assays

Cells were uniformly inoculated in cell culture plates. After 48 h of transfection, a 10 μL pipette tip was used to scratch a line at the bottom of the plates. Then, cell migration photographs were taken under a microscope at different time points (0, 24, and 48 h) after changing the cell culture medium to a serum-free medium.

### 2.8 Flow cytometry

The apoptosis rate was calculated according to the PE-Annexin-V-Apoptosis-Kit (Novozymes, Nanjing, China). In brief, the cells were isolated using EDTA-free trypsin, washed, and incubated for 10 min at 37 °C with 5 μL of annexin V-fluorescein isothiocyanate solution (FITC) containing 5 μL of propidium iodide (PI) in the dark.

Cells were harvested and fixed in 70 % ethanol for 2 h overnight at 4 °C for the cell cycle test. The cells underwent a 30 min treatment with 100 μL of RNase A in a water bath at 37 °C before being washed and stained with PI (WanleiBio, Shenyang, China) for 30 min at 4 °C. Finally, the samples were analyzed with flow cytometry (Beckman Biotechnology, Beijing, China).

### 2.9 Immunofluorescence

Immunofluorescence was used to examine the nucleation of SMAD2/3 and P-SMAD2/3. On cell slides for a 24-well plate, MGC-803 and HGC-27 cells were seeded. After fixation with 4 % paraformaldehyde and permeabilization with 0.5 % Triton X-100, the cells were respectively treated with rabbit *anti*-SMAD2/3 (1: 100 dilution) and rabbit *anti*-P-SMAD2/3 (1: 100 dilution) antibodies overnight at 4 °C. The next day, the slides were treated with a fluorescent secondary antibody (Beyotime) at room temperature for 1 h. The cell nuclei were stained with DAPI (GenePharma Suzhou, China) or Hoechst 33258 (Beyotime). A Leica fluorescence microscope was used to take pictures of the cells.

### 2.10 Immunohistochemistry (IHC)

The pathology department at Jiangsu University Hospital in Jiangsu, China, divided the tissues into sections. Endogenous peroxidase activity was suppressed using 3 % hydrogen peroxide after the tissue slices had been deparaffinized and rehydrated with ethanol. The sections were treated with the primary antibody at 4 °C overnight, incubated with anti-mouse secondary antibodies, and then visualized according to the instructions of the DAB kit (Beyotime Biotechnology, Jiangsu, China) for development.

### 2.11 Enzyme-linked immunosorbent assay (ELISA)

Experimental mouse ocular venous blood supernatants were collected. The secretion of tumor growth factor β1 (TGF-β1) and tumor necrosis factor-α (TNF-α) was calculated by spectrophotometric detection at 450 nm absorbance following the operating instructions of the mouse ELISA kit (BOSTER, Wuhan, China).

### 2.12 Luciferase reporter assay

Dual-luciferase assays validated the expected link between circMRPL35 and miR-6809-3p, miR-6809-3p, and ZNF90. Briefly, luciferase reporter gene plasmids with a wild-type sequence (WT) and with a mutation sequence in the binding region of miR-6809-3p (MUT) were generated (Genepharm). After that, luciferase reporter plasmids, miR-6809-3p mimics, or miR-NC, were cotransfected into 293T cells plated in 24-well plates. The Renilla and firefly luciferase activities were measured using the Promega system (GloMax20/20, USA) after 24 h of transfection, according to the dual luciferase reporter kit (Novozymes, Nanjing, China). Renilla luciferase activity was used to normalize luciferase activity.

### 2.13 Tissue specimens and ethical approval

A total of 37 paired stomach cancer tissues and matched adjacent tissues were collected from patients undergoing surgery at the Affiliated People's Hospital of Jiangsu University and Yixing People's Hospital between September 2020 and March 2022. The tissues were preserved at −80 °C post-collection. Patients diagnosed with primary GC by the Department of Pathology who underwent surgery without prior anticancer treatment were included. Individuals with severe mental illnesses, additional tumors, or other significant underlying conditions were excluded. This study received approval from the Ethics Committee of Jiangsu University (approval number: 2020161), and informed consent was obtained from all participants.

### 2.14 Fluorescence *in situ* hybridization (FISH) assay

To investigate the localization of circMRPL35, a FISH experiment was conducted using MGC-803 and HGC-27 cells seeded on cell slides in 24-well plates. The cells were fixed in 4 % paraformaldehyde and permeabilized with 0.1 % Triton X-100. CY3-conjugated circMRPL35 probes (5′-GTAGGATTCCTTTCTCCTCACCCAA-3′) were hybridized following the instructions of the RNA FISH kit SA-Biotin system (GenePharma, Suzhou, China). A fluorescence quenching blocker was then applied to the cell slides after staining the nuclei with DAPI (Beyotime, Shanghai, China). Finally, images were captured using a fluorescence microscope (Leica, Mannheim, Germany).

### 2.15 Animal studies

The Nanjing University Model Animal Research Center sold four-week-old male nude mice. The animal experiment was carried out in the Laboratory Animal Center of Jiangsu University following the rules of Jiangsu University and the Jiangsu Municipal Science and Technology Commission (SYXK (su) 2018-0053). All institutional and national guidelines for the care and use of laboratory animals were followed. MGC-803 cells transfected with the overexpression vector of circMRPL35 and negative control were subcutaneously injected into the rear of nude mice. After one week, we used calipers to measure the tumors' longest and shortest diameters every three days, recording them as *L* and *W*, respectively. The tumor volume was computed using *V* = *L* × *W*^2^ × 0.5. After 28 days, the ocular venous blood and tumors of the nude mice were collected for additional research.

### 2.16 Statistical analysis

Statistical analysis was performed by GraphPad Prism 9.5.0. All assays were performed in triplicate, and values are presented as mean ± SD. The χ^2^ test was calculated the data and Student's t-test analyzed the differences between the two groups. *P* < 0.05 was deemed statistically significant.

## 3 Results

### 3.1 High circMRPL35 expression in GC and enhanced tumor growth *in vivo*

**Fig. 1:**
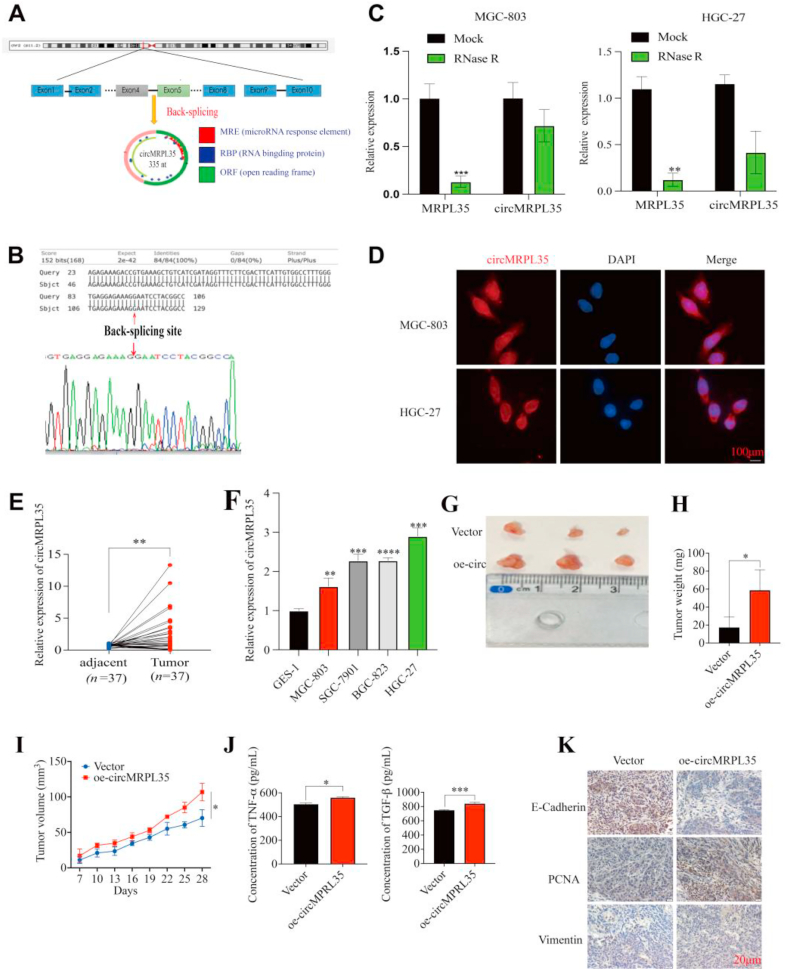
Characterization and expression of circMRPL35 in GC. **A** Schematic illustration demonstrating that the circularization of exons 4–5 of the MRPL35 gene forms circMRPL35 by the head-to-tail junction. **B** and **C** The presence of circMRPL35 was validated by Sanger sequencing and qRT-PCR after RNase R treatment. **D** The subcellular localization of circMRPL35 in MGC-803 and HGC-27 cells was confirmed by RNA FISH (scale bar = 100 μm). **E** and **F** qRT-qPCR analysis of circMRPL35 in GC tissues (**E**) and cell lines (**F**). **G** Image of nude mouse subcutaneous tumors in the control and circMRPL35-overexpressing groups. **H** Weight of subcutaneous tumors. **I** Weight of subcutaneous tumors. **J** ELISA for the detection of TGF-β and TNF-α secretion. **K** Immunohistochemical staining of subcutaneous tumor tissues (scale bar = 20 μm). Data represent the mean ± SD. ∗*P* < 0.05, ∗∗*P* < 0.01, ∗∗∗*P* < 0.001, ∗∗∗∗*P* < 0.0001.

GC circRNA expression profiles were obtained from the GEO database (GSE78092, GSE131414, and GSE100170) and analyzed by intersecting calculations. The circRNA, named circMRPL35, originated from back-splicing of the *MRPL35* on chr2 (p11.2), resulting in a 335 nt circular transcript (Fig. 1A). Sanger sequencing validated head-to-tail splicing (Fig. 1B). Divergent and convergent primers were utilized to differentiate circMRPL35 from linear MRPL35 mRNA, demonstrating circMRPL35's resistance to RNase R treatment (Fig. 1C). RNA FISH experiments showed the distribution of circMRPL35 in the cytoplasm and nucleus of MGC-803 and HGC-27 cells (Fig. 1D). qRT-PCR analysis revealed upregulation of circMRPL35 in GC tissues (Fig. 1E) and cells (Fig. 1F), correlating with lymphatic metastasis and tumor size (Table 1). HGC-27 cells exhibited higher circMRPL35 levels than MGC-803 cells, prompting their selection as cell line models for subsequent studies. To assess circMRPL35 functions *in vivo*, a xenograft mouse model was established by subcutaneously injecting MGC-803 cells transfected with circMRPL35 overexpression plasmid and control vector plasmid into nude mice. ELISA results showed elevated secretion of TGF-β1 and TNF-α upon circMRPL35 overexpression compared to the control group (Fig. 1J). These results imply that circMRPL35 could potentially function as a biomarker for GC.

**Table 1:** Correlation between circMRPL35 expression and GC clinicopathological parameters (*n* = 37).

Parameters	No.	circMRPL35 expression		*χ*^*2*^	*P* value
		High (*n* = 23)	Low (*n* = 14)		
Gender					
Male	26	14	12	2.571	0.1088
Female	11	9	2		
Age					
>65	25	16	9	0.111	0.739
≤65	12	7	5		
Tumor size (cm)					
>5	20	9	11	5.451	**0.020**
≤5	17	14	3		
LNM					
Positive	24	18	6	4.786	**0.029**
Negative	13	5	8		
TNM stages					
I + II	12	6	6	1.117	0.291
III + IV	25	17	8		

Bold values indicate statistically significant *P* values.

### 3.2 circMRPL35 knockdown impeded the GC cell phenotype, whereas circMRPL35 overexpression enhanced it *in vitro*

**Fig. 2:**
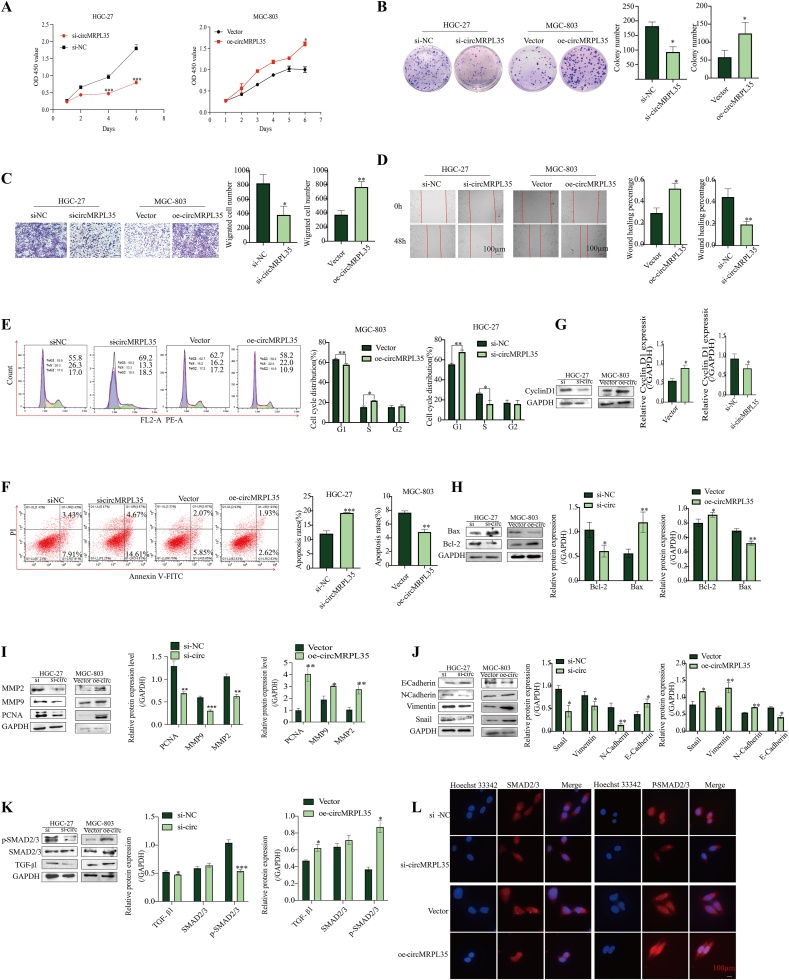
circMRPL35 promoted GC cell development *in vitro*. **A–D** CCK-8 assay (**A**), colony formation assay (**B**), transwell migration assay (**C**) and wound healing assay (**D**) were used to analyze cell proliferation and migration in transfected MGC-803 and HGC-27 cells. **E** and **F** Flow cytometry was performed to detect cell cycle progression (**E**) and apoptosis (**F**) in transfected MGC-803 and HGC-27 cells. **G–K** Western blot analysis of the expression of Cyclin D1 (**G**), Bcl-2, Bax (**H**), PCNA, MMP2, MMP9 (**I**), as well as the EMT-related proteins snail, vimentin, N-cadherin, E-cadherin (**J**) and the TGF-β1/SMAD2/3 signaling pathway-related proteins TGF-β1, SMAD2/3 and P-SMAD2/3 (**K**). **L** Immunofluorescence assay detected the expression of SMAD2/3 and P-SMAD2/3 in MGC-803 cells (scale bar = 100 μm). Data represent the mean ± SD. ∗*P* < 0.05, ∗∗*P* < 0.01, ∗∗∗*P* < 0.001.

To investigate the impact of circMRPL35 on GC cell proliferation, the cell cycle, and apoptosis, gain/loss-of-function assays were conducted. Specific siRNA for circMRPL35 and circMRPL35 overexpression plasmids were designed and transfected into MGC-803 or HGC-27 cells. The expression of circMRPL35, but not MRPL3, was manipulated within GC cells (supplementary file 2: Fig. S1a–b). Results from CCK-8, colony formation, transwell migration, cell wound healing, and flow cytometry assays indicated that circMRPL35 overexpression enhanced GC cell proliferation, mobility, G2 phase distribution, and reduced apoptosis (Fig. 2A–F). Moreover, the SMAD2/3 signaling pathway, known for its role in EMT, cell metastasis, and proliferation, was explored [16,17]. Western blot and immunofluorescence tests revealed that upregulation of circMRPL35 increased levels of various proteins related to cell cycle, apoptosis, and EMT (Fig. 2G–J) while affecting the SMAD2/3 signaling pathway (Fig. 2K–L). These findings collectively showed that circMRPL35 overexpression increases GC cell development and enhances TGF-1/SMAD2/3 and EMT signaling pathway activity *in vitro*.

### 3.3 Hsa-miR-6809-3p was a sponge of circMRPL35

**Fig. 3:**
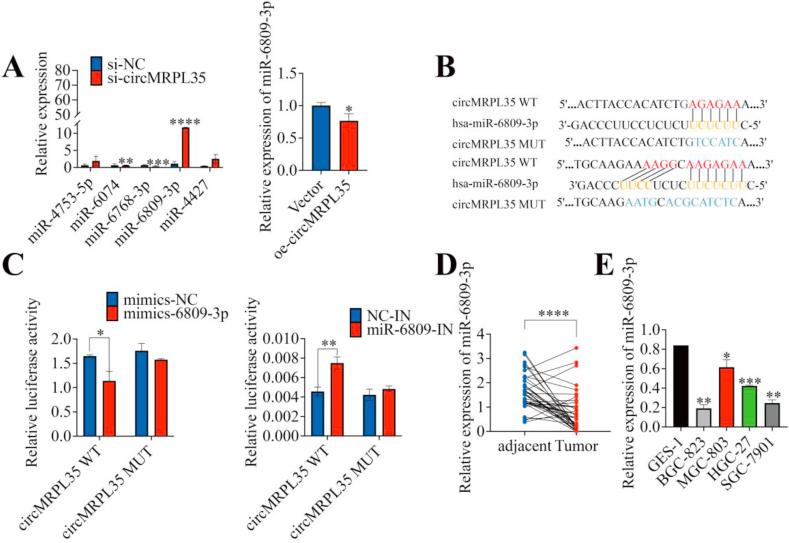
circMRPL35 sponges miR-6809-3p. **A** qRT-PCR detected the expression of potentially binding miRNAs after aberrant expression of circMRPL35. **B** Binding sites of circMRPL35 with miR-6809-3p. **C** Dual-luciferase reporter gene assay demonstrated circMRPL35 binding to miR-6809-3p. **D** and **E** qRT-PCR detected the expression of miR-6809-3p in GC tissues (**D**) and cell lines (**E**). Data represent the mean ± SD. ∗*P* < 0.05, ∗∗*P* < 0.01, ∗∗∗*P* < 0.001, ∗∗∗∗*P* < 0.0001.

Utilizing the circBank database (http://www.circbank.cn) to predict potential targets of circMRPL35 provided valuable insights into its role in GC progression. Five miRNAs with high prediction scores were initially identified as possible targets of circMRPL35, including miR-4753-5p, miR-6074, miR-6768-3p, miR-6809-3p, and miR-4427. However, experimental modulation of circMRPL35 expression indicated that only miR-6809-3p exhibited significant alterations when circMRPL35 was either silenced or overexpressed in GC cells (Fig. 3A). Next, luciferase reporter gene plasmids were constructed with the wild-type (WT) circMRPL35 sequence and a mutated (MUT) circMRPL35 sequence at the miR-6809-3p binding site using complementary pairing sequences (Fig. 3B). The dual-luciferase reporter gene assay showed that WT luciferase activity decreased with miR-6809-3p overexpression and increased with miR-6809-3p inhibition. In contrast, MUT luciferase activity remained constant (Fig. 3C), suggesting a direct targeting interaction between circMRPL35 and miR-6809-3p. Additionally, qRT-PCR analysis showed significant downregulation of miR-6809-3p in GC tissues (Fig. 3D) and cells (Fig. 3E). These findings indicate a potential regulatory role of circMRPL35 on miR-6809-3p in GC.

### 3.4 miR-6809-3p affected the GC cell phenotype

**Fig. 4:**
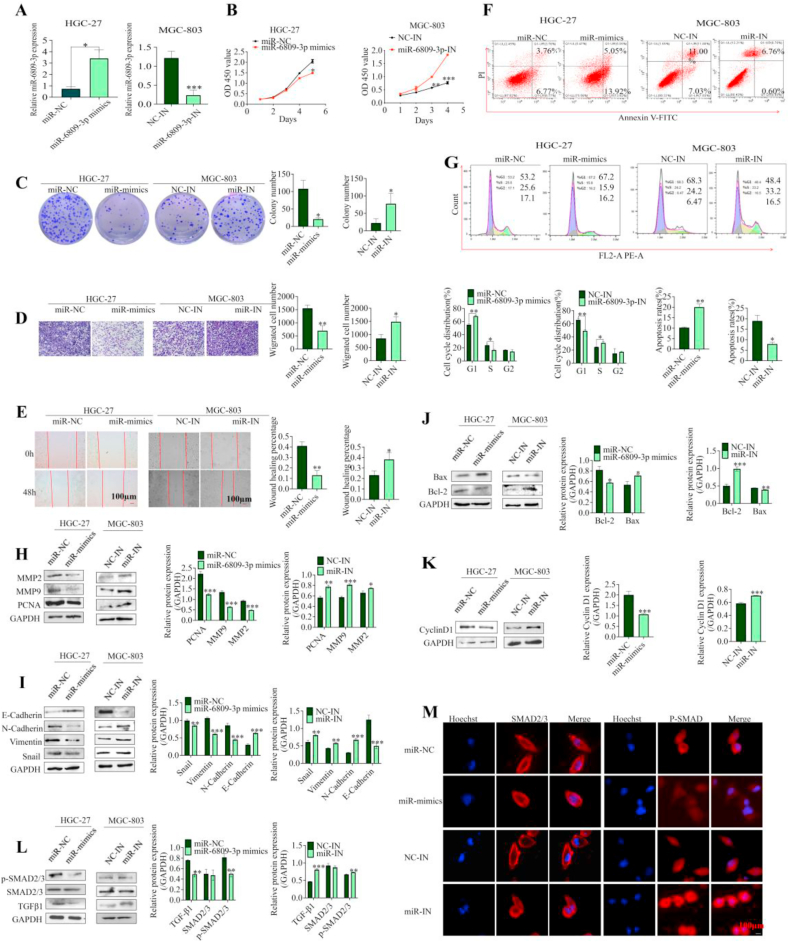
miR-6809-3p inhibits the GC cell phenotype. **A** qRT-PCR to validate the transfection efficiency in MGC-803 and HGC-27 cells. **B**–**E** CCK-8 assay (**B**), colony formation assay (**C**), transwell migration assay (**D**) and wound healing assay (**E**) were used to analyze cell proliferation and migration in transfected MGC-803 and HGC-27 cells. **F** and **G** Flow cytometry was performed to detect cell cycle progression (**F**) and apoptosis (**G**). **H**–**L** Western blot analysis of the expression of PCNA, MMP2, MMP9 (**H**), EMT-related proteins snail, vimentin, N-cadherin, E-cadherin (**I**), Cyclin D1 (**J**), Bcl-2, Bax (**K**), and TGF-β1/SMAD2/3 signaling pathway-related proteins TGF-β1, SMAD2/3 and P-SMAD2/3 (**L**). **M** Immunofluorescence assay detected the expression of SMAD2/3 and P-SMAD2/3 (scale bar = 100 μm). Data represent the mean ± SD. ∗*P* < 0.05, ∗∗*P* < 0.01, ∗∗∗*P* < 0.001.

miR-6809 has been implicated in various cancers [[18], [19], [20]], but its role in GC remains unclear. This study aimed to investigate the impact of miR-6809-3p on GC cell phenotype using overexpression and low-expression models in HGC-27 and MGC-803 cells, respectively (Fig. 4A). The results indicated that miR-6809-3p reduced the S phase of the cell cycle, induced apoptosis, and impeded the proliferative and migratory abilities of GC cells (Fig. 4B–G). Furthermore, miR-6809-3p downregulated several proteins associated with cell proliferation and migration, suggesting a suppressive effect on the GC cell phenotype (Fig. 4H–K). The study also explored the influence of miR-6809-3p on the TGF-β1/SMAD2/3 signaling pathways in GC cells, revealing a suppression of TGF-β1 and p-SMAD2/3 protein expression (Fig. 4L–M). These findings suggest that miR-6809-3p inhibits tumor growth by blocking GC cell TGF-β1/SMAD2/3 activation.

### 3.5 miR-6809-3p reverses the effect of circMRPL35 on the GC cell phenotype

**Fig. 5:**
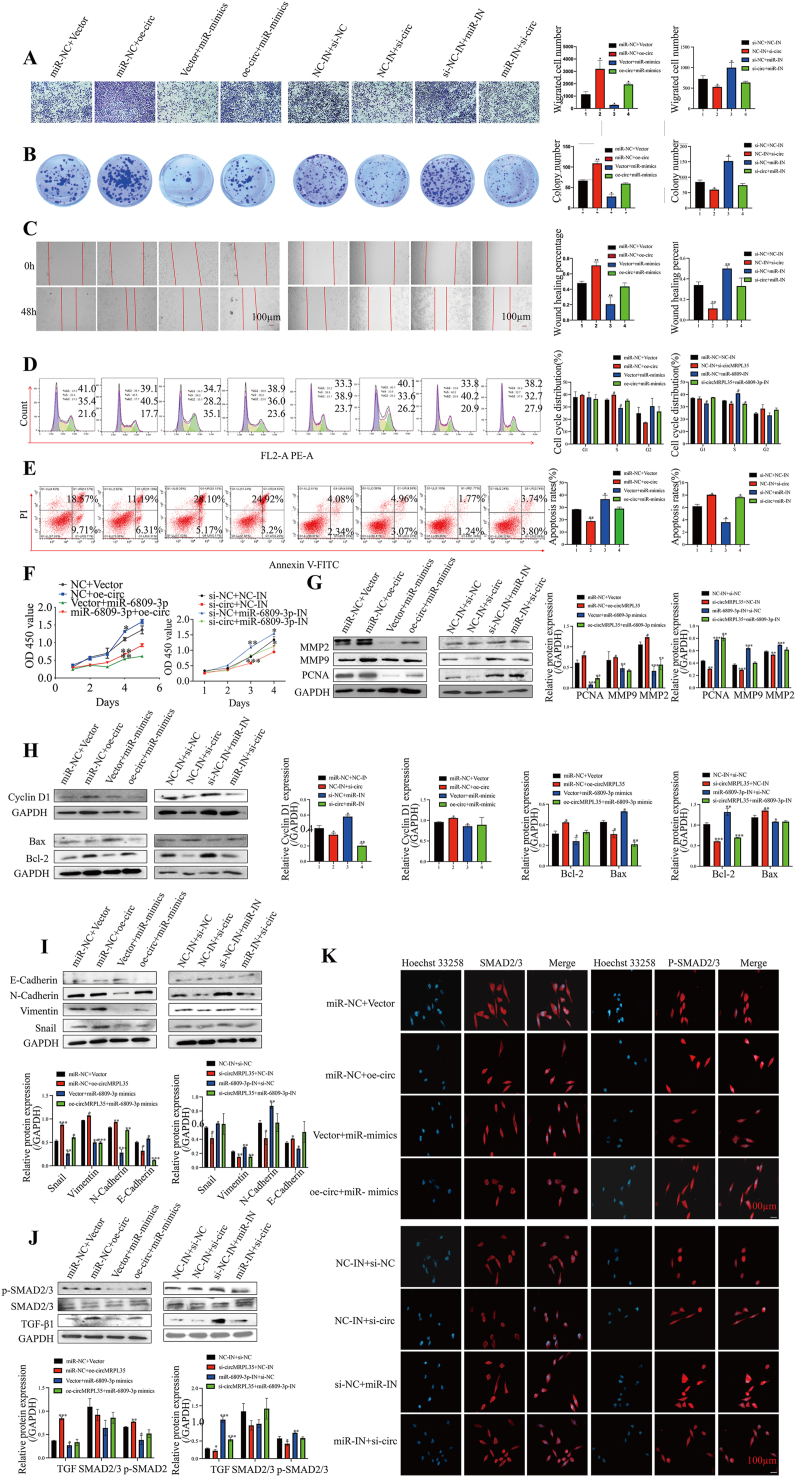
miR-6809-3p reverses the ability of circMRPL35 to inhibit the GC cell phenotype. **A–F** Transwell migration assays (**A**), colony formation assays (**B**), wound healing assays (**C**), cell cycle assays (**D**), apoptosis assays (**E**), and CCK-8 assays (**F**) were used to analyze cell proliferation, migration, cell cycle progression and apoptosis in transfected MGC-803. **G**-**J** Western blot detection of miR-6809-3p reversed the expression of proliferation-associated proteins and EMT-associated proteins caused by circMRPL35. **K** Immunofluorescence assay detected the expression of SMAD2/3 and P-SMAD2/3 (scale bar = 100 μm). Data represent the mean ± SD. ∗*P* < 0.05, ∗∗*P* < 0.01, ∗∗∗*P* < 0.001, ∗∗∗∗*P* < 0.0001.

Rescue experiments were conducted to investigate if circMRPL35 functions by sequestering miR-6809-3p through downregulation or upregulation of miR-6809-3p in cells overexpressing circMRPL35. Various assays, including Transwell, cell colony formation, wound healing, cell cycle, apoptosis, CCK-8 cell proliferation, and Western blot analyses, revealed that miR-6809-3p reversed the dysregulation of circMRPL35, leading to alterations in GC cell proliferation, migration, cell cycle, apoptosis (Fig. 5A–F), and protein expression levels (Fig. 5G–H). Additionally, miR-6809-3p counteracted the stimulatory effects of TGF-β1 and p-SMAD2/3 expression, as demonstrated by immunofluorescence and Western blot analyses (Fig. 5I–K). In conclusion, circMRPL35 sequesters miR-6809-3p to enhance the GC cell phenotype.

### 3.6 ZNF90 was a downstream target of miR-6809-3p

**Fig. 6:**
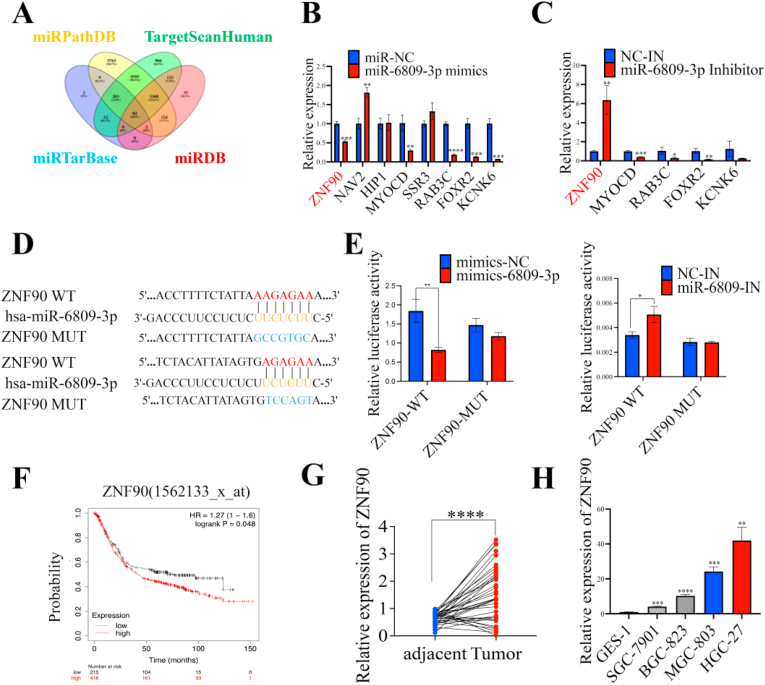
ZNF90 targets miR-6809-3p and is upregulated in GC tissues and cells. **A** Venn diagram of predicted target genes that miR-6809-3p may bind to. **B** and **C** qRT‒PCR analysis of ZNF90 expression downregulation (**B**) and upregulation (**C**) after miR-6809-3p overexpression and inhibition. (**D**) Binding sites of miR-6809-3p and ZNF90. **E** Dual-luciferase reporter gene assay verified direct binding between ZNF90 and miR-6809-3p. **F** Survival curve of ZNF90 in GC. **G** and **H** ZNF90 was upregulated in GC tissues (**G**) and cell lines (**H**). Data represent the mean ± SD. ∗*P* < 0.05, ∗∗*P* < 0.01, ∗∗∗*P* < 0.001, ∗∗∗∗*P* < 0.0001.

Utilizing information from miRPathDB (https://mpd.bioinf.uni-sb.de/overview.html), TargetScan (https://www.targetscan.org/vert_72/), miRTarBase (http://mirtarbase.mbc.nctu.edu.tw/php/index.php), and miRDB (http://mirdb.org) databases, a Venn diagram was created to pinpoint potential target genes of miR-6809-3p (Fig. 6A). Following qPCR experiments targeted candidates with high binding site scores and established oncogenic functions, informed by literature scrutiny. Findings indicated that alterations in miR-6809-3p expression had an inverse impact on ZNF90 levels, validated through qRT-PCR (Fig. 6B–C), Western blot, IHC, qRT-PCR, and immunofluorescence assessments in murine tissues and GC cells (supplementary file 2: Fig. S2a–e). Dual-luciferase reporter gene assays employing wild-type (WT) and mutant (MUT) ZNF90 3′-UTR sequences confirmed the direct interaction between miR-6809-3p and ZNF90 (Fig. 6D–E). Additionally, elevated ZNF90 expression was associated with reduced overall survival in GC patients (Fig. 6F) and exhibited upregulation in GC tissues and cells relative to controls (Fig. 6G–H). These findings substantiate the hypothesis that miR-6809-3p modulates ZNF90 mRNA to control its expression, thereby identifying ZNF90 as a downstream mediator of miR-6809-3p.

### 3.7 ZNF90 affects the GC cell phenotype and promotes TGF-β1/Smad2/3 signaling pathways

**Fig. 7:**
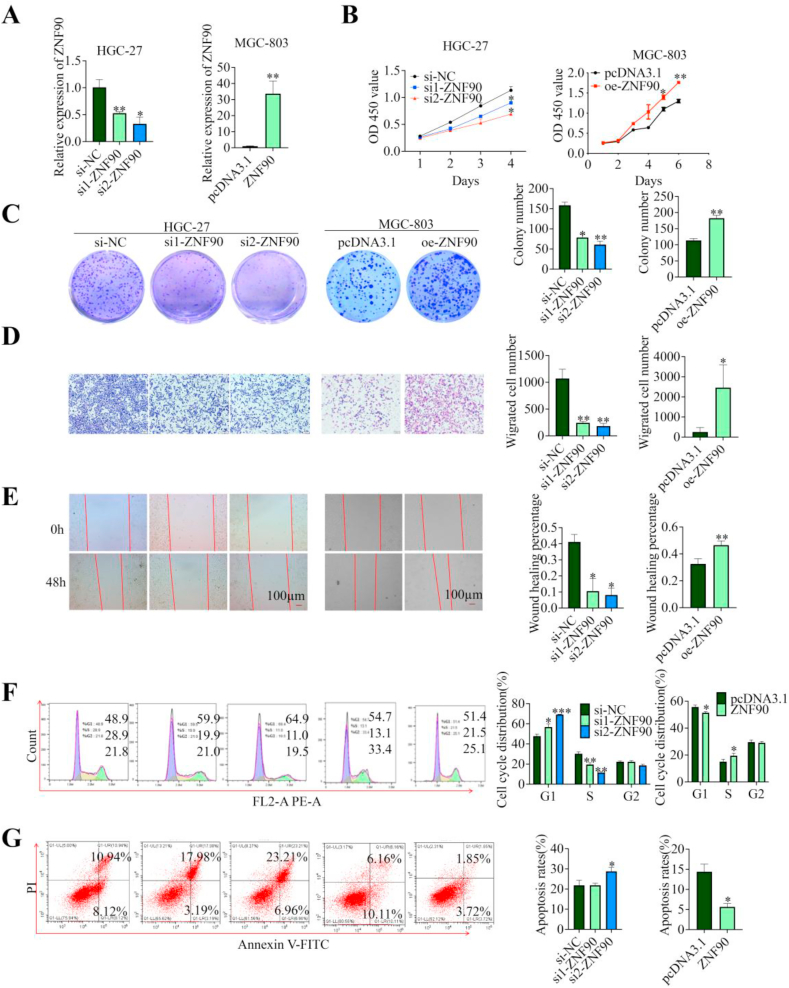
ZNF90 promotes the GC cell phenotype and TGF-β1/Smad2/3 signaling pathways. **A** qRT-PCR to validate the transfection efficiency in MGC-803 and HGC-27 cells. **B–E** CCK-8 assay (**B**), colony formation assay (**C**), transwell migration assay (**D**) and wound healing assay (**E**) revealed that ZNF90 promoted cell proliferation and migration in transfected MGC-803 and HGC-27 cells. **F** and **G** Flow cytometry demonstrated that ZNF90 promoted cell cycle progression (**F**) and inhibited apoptosis (**G**). Data represent the mean ± SD. ∗*P* < 0.05, ∗∗*P* < 0.01, ∗∗∗*P* < 0.001.

The biological functions of ZNF90 were investigated due to a lack of studies on its role in GC. Following efficient repression and overexpression of ZNF90 in GC cells (Fig. 7A), a series of cell function experiments showed that ZNF90 overexpression increased GC cell mobility and migration, led to more GC cells entering the G2 phase, and reduced apoptosis (Fig. 7B–G, Additional file 2: Fig. S3a–c). Moreover, Immunofluorescence and Western blot results also indicated that ZNF90 enhanced the intracellular expression p-SMAD2/3 and facilitated the EMT process (Additional file 2: Fig. S3d–f). In summary, our findings suggest that ZNF90 may promote the development of GC cells *in vitro*.

### 3.8 miR-6809-3p weakens the phenotypes of GC cells by targeting ZNF90, thus further affecting the TGF-β1/SMAD2/3 signaling pathway

**Fig. 8:**
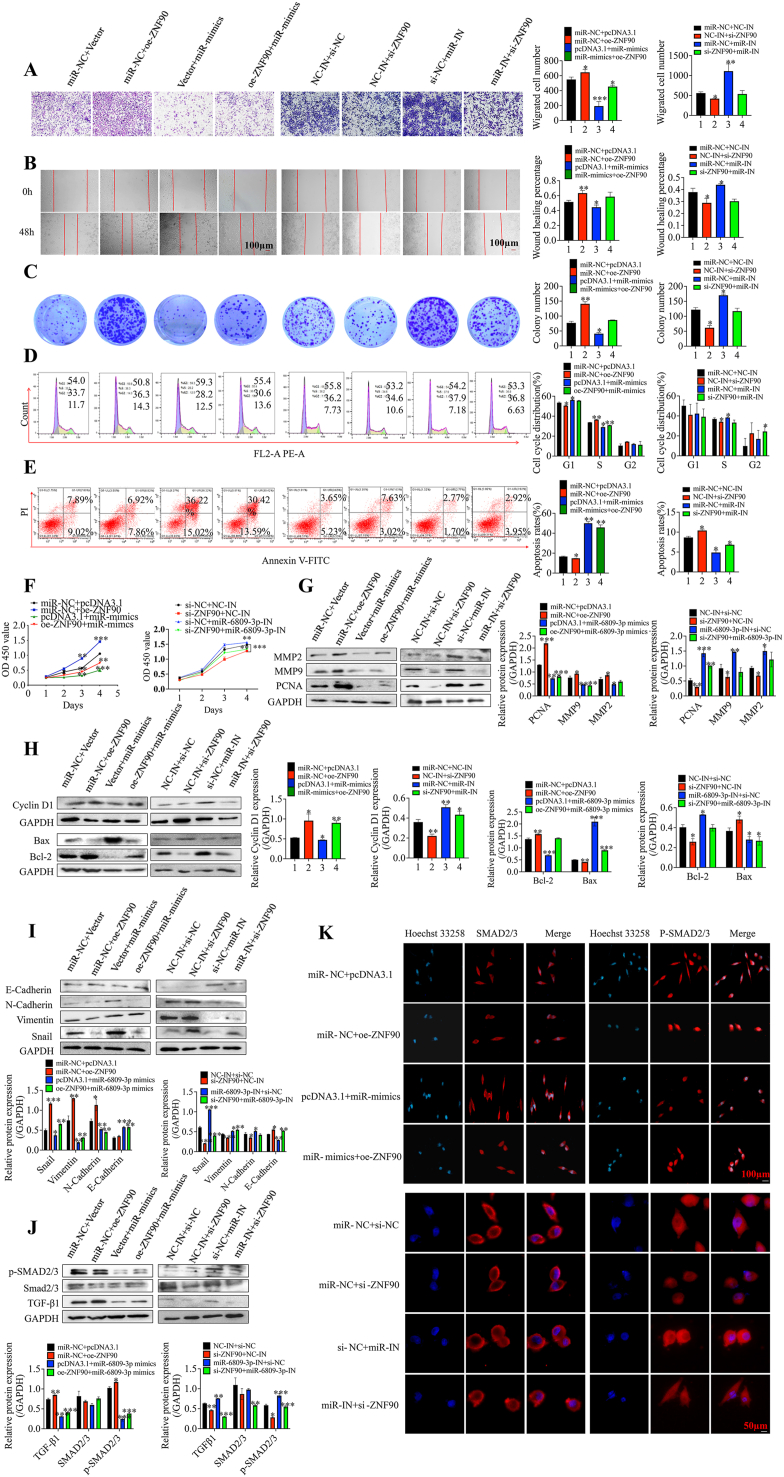
miR-6809-3p reverses the ability of ZNF90 to inhibit the GC cell phenotype and the TGF-β/SMAD2/3 signaling pathway. **A–F** Transwell migration assays (**A**), wound healing assays (**B**), colony formation assays (**C**), cell cycle assays (**D**), apoptosis assays (**E**), and CCK-8 assays (**F**) were used to analyze whether miR-6809-3p reversed the effects of ZNF90 on cell proliferation, migration, cell cycle progression and apoptosis in transfected MGC-803. **G–J** Western blot detection of miR-6809-3p reversed the expression of proliferation-associated proteins and EMT-associated proteins caused by ZNF90. **K** Immunofluorescence assay detected the expression of SMAD2/3 and P-SMAD2/3 (scale bar = 100 μm). Data represent the mean ± SD. ∗*P* < 0.05, ∗∗*P* < 0.01, ∗∗∗*P* < 0.001.

Rescue tests were conducted to investigate the impact of miR-6809-3p on ZNF90 functions in GC development. Results from the CCK-8 assay, cloning assay, transwell migration, and cell wound scratch healing tests indicated that miR-6809-3p partially reversed the increased proliferation and migratory capacity induced by ZNF90 in MGC-803 cells (Fig. 8A–C, F). Additionally, Western blot experiments and flow cytometry analysis suggested that miR-6809-3p could partially restore ZNF90-induced changes in cell cycle distribution and apoptosis rates (Fig. 8D–E, H). Further, Western blot tests and immunofluorescence assays revealed that miR-6809-3p partially reversed ZNF90-induced alterations in the expression levels of associated proteins and the activation of epithelial-mesenchymal transition (EMT) and the TGF-β1/SMAD2/3 signaling pathway (Fig. 8I–K). These results suggest that miR-6809-3p regulates GC cell phenotype by modulating ZNF90 expression to attenuate TGF-β1/SMAD2/3 signaling pathway activity.

## 4 Discussion

A novel family of abundant and highly stable single-stranded circular RNAs, circRNAs, has been discovered [21]. Advances in bioinformatics and high-throughput sequencing technology have spurred growing research showcasing the significant roles circRNAs play in cancer initiation and advancement [22]. They also hold promise as diagnostic, prognostic, and predictive biomarkers. However, the current understanding of circRNAs linked to GC is restricted, with limited documented functions and mechanisms. Most circRNAs still necessitate additional exploration of their biological functions.

This study identified a circRNA called circMRPL35, exhibiting upregulation in GC tissues and cells and correlating with lymph node metastasis and increased tumor size. Functionally, circMRPL35 enhanced GC cell proliferation, migration, and G1/S phase transition of the cell cycle, inhibited apoptosis, and facilitated *in vivo* tumor growth. Research has suggested that during metastasis, EMT entails the transformation of mesenchymal cells into a metastatic phenotype [23]. This transformation is marked by the reduction of E-cadherin and the acquisition of Vimentin and N-cadherin, constituting the fundamental mechanism of tumor cell metastasis [24,25]. In GC invasion and metastasis, matrix metalloproteinases (MMPs) are recognized as pivotal contributors to cell proliferation and metastasis [[26], [27], [28]]^.^ Recent studies have emphasized the engagement of the TGF-β/SMAD signaling pathway in tumor metastasis, apoptosis, and the EMT process [16,29,30]. Phosphorylation of SMAD2 and SMAD3 in the cytoplasm triggers the formation of a complex with SMAD4, which subsequently translocates to the nucleus to commence gene transcription [31]. This process partially controls the expression of EMT-related proteins, leading to increased Vimentin and N-cadherin transcripts and decreased E-cadherin transcripts [32]. These results imply that the TGF-β1/SMAD2/3 pathway could serve as a potential inducer of EMT. The present study shows that circMRPL35 boosts the expression of proteins like PCNA, MMP2, MMP9, and other factors related to apoptosis, cell cycle regulation, EMT, and TGF-β1 and p-SMAD2/3. These findings suggest that circMRPL35 might facilitate EMT by amplifying the TGF-β1/SMAD2/3 signaling pathway, thereby impacting GC cells' proliferation, migration, and apoptosis.

CircRNAs contain specific sequences called miRNA response elements (MREs) that act as decoys for miRNAs, modulating the expression of target genes. Activating signaling pathways is one established mechanism by which circRNAs contribute to cancer progression [11,33]. For example, hsa_circ_0009910 promotes ovarian cancer cell proliferation by sequestering miR-145, activating NF-κB and Notch signaling pathways [34]. Similarly, CircANKS1B promotes breast cancer invasion and metastasis by sequestering miR-148a-3p and miR-152-3p, leading to the upregulation of the transcription factor USF1 and a subsequent increase in TGF-β1 expression, ultimately activating the TGF-β1/SMAD signaling cascade to induce EMT [35]. Conversely, circ-OXCT1 inhibits EMT and metastasis in GC by blocking the TGF-β/SMAD signaling pathway via the miR-136/SMAD4 axis [36]. Our bioinformatics analysis indicated that circMRPL35 and ZNF90 possess the MRE for miR-6809-3p, hinting at forming a circMRPL35/miR-6809-3p/ZNF90 regulatory axis. Subsequent luciferase reporter assays confirmed that circMRPL35 functions as a sponge for miR-6809-3p, suppressing ZNF90 expression by binding to the 3′-UTR of ZNF90 mRNA. Due to the unknown functions of miR-6809-3p and ZNF90 in GC, this study further explores their expression profiles and biological roles. Initially, the study noted a reduction in miR-6809-3p levels in GC tissues and cell lines, suppressing GC cell migration and proliferation, enhancement of apoptosis, and influencing EMT and the TGF-β1/SMAD2/3 pathway. In contrast, ZNF90 expression was elevated in GC tissues and cell lines, acting as an oncogene that promoted the GC cell phenotype. Additionally, miR-6809-3p could counter the impacts on migration, proliferation, apoptosis, EMT, and the TGF-β1/SMAD2/3 pathway in GC cells caused by aberrant circMRPL35 and ZNF90 levels. Based on these results, we infer that circMRPL35 plays a role in GC advancement by regulating the miR-6809-3p/ZNF90 axis. Although circMRPL35 adds to this growing body of knowledge, its specific functions and regulatory mechanisms warrant further investigation to fully understand its relative novelty and potential as a therapeutic target.

This study has several limitations. Although we demonstrated that circMRPL35 is highly expressed in gastric cancer cells, its transfer via extracellular vesicles (EVs) and influence on distant tissues remain unconfirmed. Previous research shows that exosomal miR-9-5p promotes liver metastasis by regulating cholesterol enzymes, and inhibiting this miRNA can prevent metastasis [37]. Additionally, over 136,000 EV-associated circRNAs have been identified as potential biomarkers, with YBX1 mediating their packaging into EVs [38], indicating that EVs are rich in circular RNAs involved in tumor progression. Future investigations should isolate EVs from gastric cancer cells and patient samples to verify circMRPL35 packaging and its role in intercellular communication and metastasis. Furthermore, although the circMRPL35/miR-6809-3p/ZNF90 axis influences the TGF-β1/SMAD2/3 signaling pathway, the mechanisms by which this axis affects SMAD nuclear translocation and transcriptional regulation were not explored and warrant further study. Additionally, as part of the broader landscape of circRNAs associated with gastric cancer, the regulatory mechanisms and therapeutic potential of circMRPL35 require further elucidation. Comparative analyses with other tumor-associated circRNAs could help clarify its unique role within this regulatory network. Tumor metastasis critically impacts patient survival and quality of life; thus, preventing GC metastases is a promising strategy. However, we could not evaluate circMRPL35's role in metastasis *in vivo* or its clinical relevance due to experimental limitations. Our data also indicate that circMRPL35 is expressed in both the cytoplasm and nucleus, potentially regulating RNA-binding proteins and protein synthesis, as supported by RNA FISH and bioinformatics analyses. Further research is necessary to explore these functions and their implications for gastric cancer progression.

## 5 Conclusion

In conclusion, our research indicates that circMRPL35 potentiates the TGF-β1/SMAD2/3 signaling cascade and subsequent EMT by upregulating ZNF90 expression through the sequestration of miR-6809-3p. This mechanism facilitates GC progression, suggesting a potential therapeutic target.

## CRediT authorship contribution statement

**Xiuping Wang:** Writing – review & editing, Writing – original draft, Supervision, Investigation, Funding acquisition, Data curation. **Zhendong Yao:** Writing – review & editing, Writing – original draft, Supervision. **Yu Liu:** Writing – review & editing, Writing – original draft. **Boneng Mao:** Writing – review & editing, Writing – original draft, Supervision. **Chen Shao:** Writing – review & editing, Writing – original draft, Supervision. **Shihe Shao:** Writing – review & editing, Writing – original draft, Supervision, Funding acquisition.

## Ethics approval

This study was approved by the Ethics Committee of the School of Medicine, Jiangsu University (Approval Number: 2020161). The animal experiments in this research were conducted in the animal center of Jiangsu University (license number: SYXK (su) 2018-0053). All institutional and national guidelines for the care and use of laboratory animals were followed.

## Consent for publication

Patient participation in the study was voluntary, and all patients signed consent forms, including consent to publish.

## Data availability statement

All data presented in this article are available from the author and can be provided upon reasonable request.

## Funding

This work was supported by grants from the 10.13039/501100001809National Natural Science Foundation of China [No. 81772157], the Jiangsu Provincial Key Laboratory of Laboratory Medicine Open Fund Project [Grant number JSKLM-Y-2024-015], and Kunshan First People's Hospital Healthcare Science and Technology Innovation Special Project [Grant number KETDCX202431].

## Declaration of competing interest

The authors declare that they have no known competing financial interests or personal relationships that could have appeared to influence the work reported in this paper.
