# Supplementary material for: circMRPL35 promotes gastric cancer progression through the miR-6809-3p/ZNF90 axis and affects the EMT process and TGF-β1/SMAD2/3 signaling
Source: Noncoding RNA Res. 2025 Oct 10;16:79–92. doi: 10.1016/j.ncrna.2025.10.002 (PMC12603770; doi:10.1016/j.ncrna.2025.10.002)
Supplement: Multimedia component 1 [file mmc1.docx]

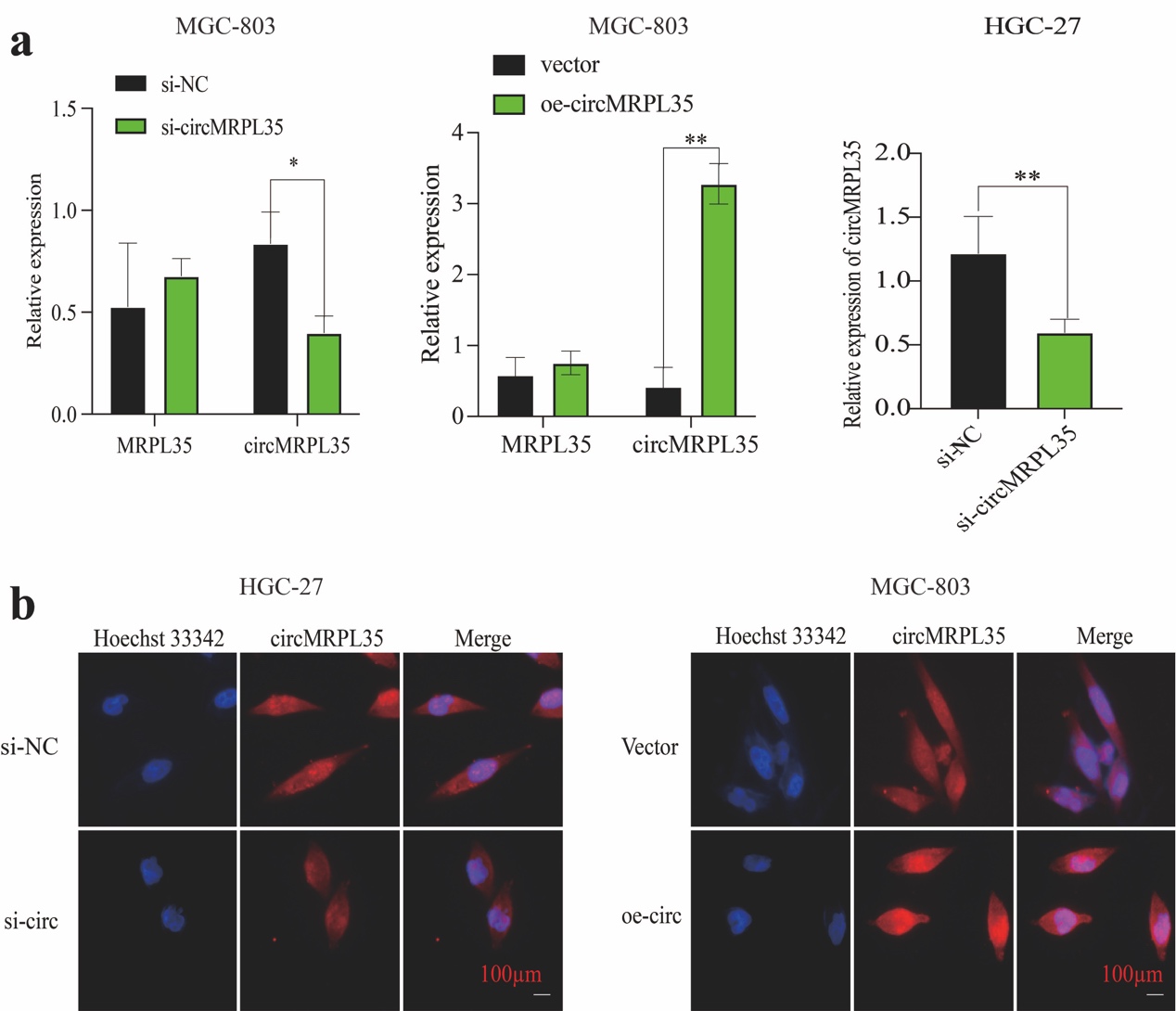


**Fig. S1** circMRPL35 promoted GC cell development in vitro. **a** The presence of circMRPL35 was validated by Sanger qRT‒PCR after RNAse R treatment. **b** RNA FISH (scale bar = 100 μm) was performed to validated the transfection efficiency in MGC-803 and HGC-27 cell. Data represent the means±SD. **P*<0.05, ***P*<0.01.


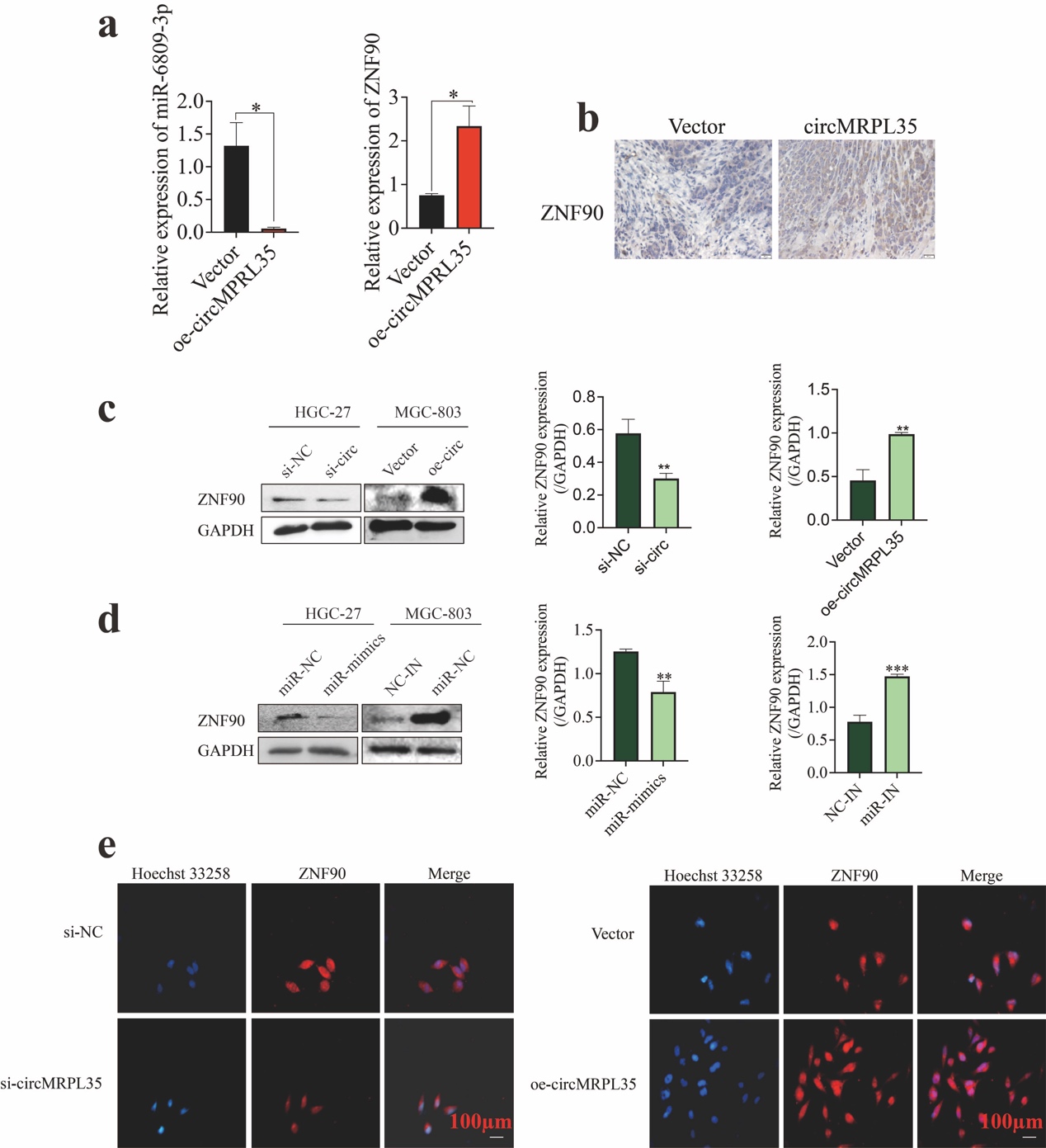


**Fig. S2** ZNF90 directly targets miR-6809-3p and is upregulated in GC tissues and cells. a. qRT-PCR analysis of the expression of miR-6809-3p and ZNF90 in nude mouse tissues; b Immunohistochemical staining of subcutaneous tumor tissues (scale bar = 20 μm), c and d western blot analysis of the expression of ZNF90 in gastric cancer cells, e Immunofluorescence assay detected the expression of ZNF90 in MGC-803 cells (scale bar = 100 μm). Data represent the means±SD. **P*<0.05, ***P*<0.01, ****P*<0.001.


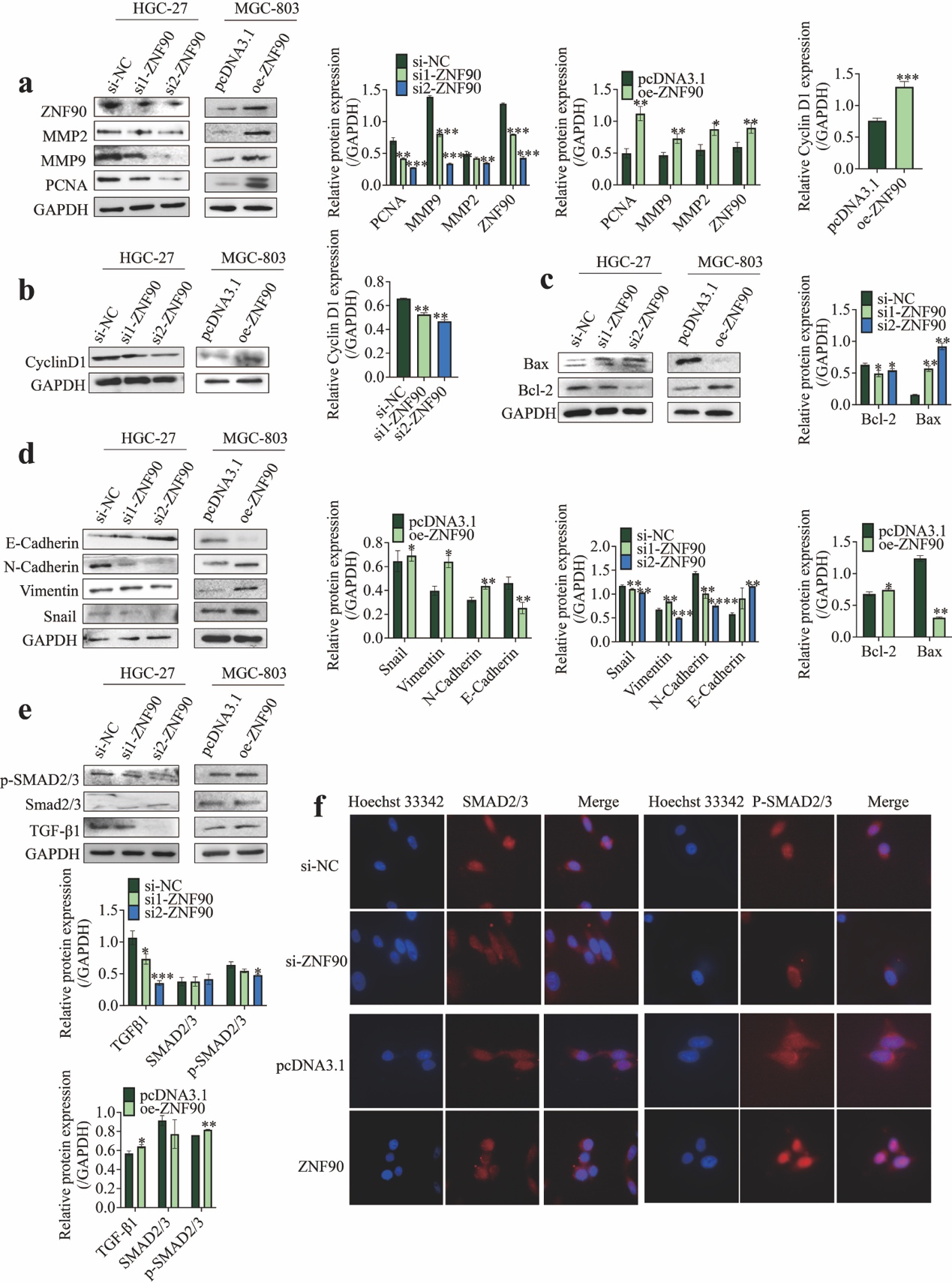


**Fig. S3** ZNF90 promotes the GC cell phenotype and TGF-β1/Smad2/3 signaling pathways. **a** and **e** Western blot analysis of the expression of ZNF90, PCNA, MMP2, MMP9 (**a**), Cyclin D1 (**b**), Bcl-2, Bax (**c**), EMT-related proteins snail, vimentin, N-cadherin, E-cadherin (**d**), and TGF-β1/SMAD2/3 signaling pathway-related proteins TGF-β1, SMAD2/3 and P-SMAD2/3 (**e**). **f** Immunofluorescence assay detected the expression of SMAD2/3 and P-SMAD2/3 (scale bar = 50 μm). Data represent the means±SD. **P*<0.05, ***P*<0.01, ***P*<0.001, **P*<0.0001.
