# Supplementary material for: circMRPL35 promotes gastric cancer progression through the miR-6809-3p/ZNF90 axis and affects the EMT process and TGF-β1/SMAD2/3 signaling
Source: Noncoding RNA Res. 2025 Oct 10;16:79–92. doi: 10.1016/j.ncrna.2025.10.002 (PMC12603770; doi:10.1016/j.ncrna.2025.10.002)
Supplement: Multimedia component 2 [file mmc2.docx]

**Methods**

**Western blot**

Total proteins were extracted using radioimmunoprecipitation assay buffer (RIPA) supplemented with 1% phenylmethanesulfonyl fluoride (PMSF; Beyotime), followed by separation using SDS-PAGE and transfer onto polyvinylidene fluoride (PVDF) membranes (Millipore, MA, USA). Subsequently, the PVDF membranes were blocked with 5% skim milk for 2 hours, incubated overnight at 4 °C with the respective primary antibodies, and then incubated for 1 h at room temperature with secondary antibodies. The Image Quant LAS 4000 mini (Pittsburgh, PA, USA) enhanced chemiluminescence (ECL) system was employed for membrane analysis.

**Transfection of oligonucleotides and plasmids**

Oligonucleotides (GenePharma) and plasmids (Sangon Biotech, Shanghai, China) were designed and produced to modulate the expression of circMRPL35, miR-6809-3p, and ZNF90. The oligonucleotides and plasmids were transfected into cells using Lipofectamine 8000 (Beyotime, Shanghai, China) following the manufacturer's instructions. The specific sequences can be found in Table S1.

**RNA isolation, RNase R treatment, and quantitative real-time PCR (qRT‒PCR)**

Total RNA was extracted using TRIzol (Vazyme, Nanjing, China) following the manufacturer's protocol. To treat with RNase R, 10 μg of total RNA was incubated with 20 U of RNase R (BioVision, Palo Alto, USA) at 37 °C for 15 minutes. To treat with RNase R, 10 μg of total RNA was incubated with 20 U of RNase R (BioVision, Palo Alto, USA) at 37 °C for 15 minutes. cDNA was synthesized from RNA through reverse transcription with HiScript® III 1st St (Vazyme). Relative expression levels were determined using the 2-ΔΔCt method, with GAPDH or U6 as the internal controls. Specific primer details can be found in Table S2.

**Cell Counting Kit-8 (CCK-8) proliferation and colony formation assay**

To evaluate the viability of the transfected cells, the CCK-8 test was performed. Specifically, 96-well plates were utilized, with 1000 cells per well and three duplicates for each treatment. The optical density was assessed using a microplate reader (Biotek, USA), with absorbance readings taken at 450 nm. For the colony formation assay, six-well plates were used, each with three replicates and approximately 1000 transfected cells per well. Following a two-week incubation period, the cell clones were fixed using 4% paraformaldehyde and then stained with crystal violet (Chemical Reagent, Nanjing, China).

**Transwell migration assay**

The transwell upper chambers (pore size, 8 m; Bio-Rad, CA, USA) were seeded with 1×10^5^ transfected cells in 300 µL serum-free media, and the bottom chambers were filled with 600 µL fresh medium containing 10% FBS. Transwell top chambers were cultured for 24 hours before being cleaned with phosphate-buffered saline (PBS), fixed with 4% paraformaldehyde, and stained with crystal violet. Finally, the cells were counted and photographed under a microscope.

**Cell wound healing assays**

Cells were uniformly inoculated in cell culture plates. After 48 h of transfection, a 10 µL pipette tip was used to scratch a line at the bottom of the plates. Then, cell migration photographs were taken under a microscope at different time points (0, 24 and 48 h) after changing the cell culture medium to a serum-free medium.

**Flow cytometry**

According to the PE-Annexin-V-Apoptosis-Kit (Novozymes, Nanjing, China), the apoptosis rate was calculated. In brief, the cells were isolated using EDTA-free trypsin,

washed and incubated for 10 min at 37°C with 5 µL of annexin V-fluorescein isothiocyanate solution (FITC) containing 5 µL of propidium iodide (PI) in the dark.

For the cell cycle test, cells were harvested and fixed in 70% ethanol for 2 hours overnight at 4 °C. The cells underwent a 30 min treatment with 100 µL of RNase A in a water bath at 37 °C before being washed and stained with PI (WanleiBio, Shenyang, China) for 30 min at 4 °C. Finally, the samples were analyzed with flow cytometry (Beckman Biotechnology, Beijing, China).

**Immunofluorescence**

To examine the nucleation of SMAD2/3 and P-SMAD2/3, immunofluorescence was used. On cell slides for a 24-well plate, MGC-803 and HGC-27 cells were seeded. After fixation with 4% paraformaldehyde and permeabilization with 0.5% Triton X-100, the cells were respectively treated with rabbit anti-SMAD2/3 (1: 100 dilution) and rabbit anti-P-SMAD2/3 (1: 100 dilution) antibodies overnight at 4°C. The next day, the slides were treated with a fluorescent secondary antibody (Beyotime) at room temperature for 1 h. The cell nuclei were stained with DAPI (GenePharma Suzhou, China) or Hoechst 33258 (Beyotime). A Leica fluorescence microscope was used to take pictures of the cells.

**Immunohistochemistry (IHC)**

The pathology department at Jiangsu University Hospital in Jiangsu, China, divided the tissues into sections. Endogenous peroxidase activity was suppressed using 3% hydrogen peroxide after the tissue slices had been deparaffinized and rehydrated with ethanol. The sections were treated with the primary antibody at 4°C overnight, incubated with anti-mouse secondary antibodies and then visualized according to the instructions of the DAB kit (Beyotime Biotechnology, Jiangsu, China) for development.

**Enzyme-linked immunosorbent assay (ELISA)**

Experimental mouse ocular venous blood supernatants were collected. The secretion of tumor growth factor β1 (TGF-β1) and tumor necrosis factor-α (TNF-α) was calculated by spectrophotometric detection at 450 nm absorbance following the operating instructions of the mouse ELISA kit (BOSTER, Wuhan, China).

**Luciferase reporter assay**

Dual-luciferase assays were utilized to validate the expected link between circMRPL35 and miR-6809-3p, as well as miR-6809-3p and ZNF90. Briefly, luciferase reporter gene plasmids with a wild-type sequence (WT) and with a mutation sequence in the binding region of miR-6809-3p (MUT) were generated (Genepharm). After that, luciferase reporter plasmids, miR-6809-3p mimics, or miR-NC were cotransfected into 293T cells that had been plated in 24-well plates. The Renilla and firefly luciferase activities were measured using the Promega system (GloMax20/20, USA) after 24 hours of transfection, according to the dual luciferase reporter kit (Novozymes, Nanjing, China). Renilla luciferase activity was used to normalize luciferase activity.

Table S1. The sequences of primers for qRT-PCR.

| Gene |  | Sequences（5’-3’） |
| --- | --- | --- |
| CircMRPL35 | Sense  Antisense | CTTTGGGTGAGGAG  AGGCATTCTTGACACAGTTGCG |
| MRPL35 | Sense  Antisense | CCTGCAAGGAAGAAGCGATTG  AATGGGGGAGTGCCAGTTTC |
| miR-6809-3p | Sense  Antisense | GCGCGCTTCTCTTCTCTCCT  AGTGCAGGGTCCGAGGTATT |
| ZNF90 | Sense  Antisense | TGGTCTTCCTTGGTATTGTTGT  TGGCAATCATCTCATGTCTCTT |
| U6 | Sense  Antisense | CTCGCTTCGGCAGCACA  AACGCTTCACGAATTTGCGT |
| GAPDH | Sense  Antisense | GGGAAGGTGAAGGTCGGAGT  GGGGTCATTGATGGCAACA |

Table S2. The sequences of primers for transfection

| Gene |  | Sequences（5’-3’） |
| --- | --- | --- |
| CircMRPL35 siRNA | Sense  Antisense | GUGAGGAGAAAGGAAUCCUTT  AGGAUUCCUUUCUCCUCACTT |
| miR-6809-3p mimics | Sense  Antisense | CUUCUCUUCUCUCCUUCCCAG  GGGAAGGAGAGAAGAGAAGUU |
| miR-6809-3p inhibitor |  | CUGGGAAGGAGAGAAGAGAAG |
| ZNF90 siRNA1 | Sense  Antisense | GGCAGAGCAUUUAUUUCAUTT  AUGAAAUAAAUGCUCUGCCTT |
| ZNF90 siRNA2 | Sense  Antisense | GCCCUUAGCACACAUAAGATT  UCUUAUGUGUGCUAAGGGCTT |
